# Self-Management Embedded in Daily Activities: A Photoelicitation Focus Group Study among Persons with Spinal Cord Injury and Their Primary Caregivers in Bangladesh

**DOI:** 10.1155/2022/2705104

**Published:** 2022-03-15

**Authors:** Salma Begum, Yeasir A. Alve, Peter Bontje

**Affiliations:** ^1^Department of Occupational Therapy, Graduate School of Human Health Sciences, Tokyo Metropolitan University, Tokyo, Japan; ^2^Department of Occupational Therapy, UIC College of Applied Health Sciences, Chicago, IL, USA

## Abstract

**Purpose:**

This study explored how community-dwelling persons with spinal cord injury (SCI) and their primary caregivers execute self-management strategies in daily activities. These strategies were mapped to a preexisting self-management framework.

**Methods:**

Photoelicitation focus group discussions were conducted among 14 adults with SCI and their primary caregivers (in two groups). Moreover, a constant comparative framework was used to analyze the data.

**Results:**

This study identified nine groups of self-management strategies, some of which could not be categorized under the three main self-management components generally accepted in the literature. Accordingly, a new component is proposed based off of this analysis, entitled management of social complexities, which includes crucial strategies such as (1) relocating to another environment, (2) behaving in an assertive manner, and (3) advocating for social change.

**Conclusion:**

The results show that self-management, traditionally described as medical, emotional, and role management, should also include the management of social complexities. The identified strategies could be considered in the development of self-management enhancement programs in lower-middle-income countries.

## 1. Introduction

Spinal cord injury (SCI) can result in lifelong disability and can severely reduce an individual's quality of life due to impairments in physical, psychological, and social functioning [1, 2]. In lower-middle-income countries (LMICs), such as Bangladesh, SCI comes with high levels of morbidity due to secondary complications, particularly pressure ulcers, pain, urinary complications, and bowel complications [3]. These factors result in 5- and 10-year survival rates of only 50% and 15%, respectively [4, 5]. Lack of financial resources and knowledge of proper care among persons with SCI, their families, and people in their communities, combined with a lack of affordable healthcare, largely contribute to this sad state of affairs [6]. Moreover, decreased social acceptance, increased stigma, and ongoing discrimination further impede persons with SCI in Bangladesh from achieving a good quality of life in society, and many lose their volition for social participation and their work lives [7, 8].

Empowering persons with SCI (in collaboration with primary caregivers) [9] to manage their health and occupational lives is necessary, given that limited specialty services and detrimental social conditions are unlikely to improve in a timely manner. In such conditions, a recent scoping review into facilitators of and constraints to living in the community for persons with SCI highlighted the need for, among other issues, development of innovative support services that empower persons living with spinal cord injury to enhance their daily lives and health [10]. Poor survival rates and severe constraints to participation in productive activities and community life lead to the question of how these individuals could be empowered to manage their health and daily activities. In recent years, self-management has gained attention as a critical prevention strategy for averting the development and recurrence of secondary complications [11] and may also contribute to increased functional performance and quality of life [12].

Self-management has been defined as “an individual's ability, in conjunction with family, community, and appropriate health professionals, to manage the symptoms, treatment, physical, and psychosocial consequences and lifestyle changes inherent in living with a chronic condition” [[13], p.1145]. Moreover, in a seminal report, Corbin and Strauss defined three domains of self-management: (a) medical management, which deals with the physical health consequences; (b) emotional management, which refers to coping with the emotions and lifestyle changes associated with the disease; and (c) role management, which refers to the way people continue their lives and regain and maintain roles [14]. Self-management programs can improve health outcomes for individuals with SCI by decreasing hospital readmissions, reducing secondary complications, improving social participation, and enhancing self-efficacy, quality of life, and well-being [15–19]. Such outcomes are in spite of the tendency of health promotion and patient self-management programs for persons with SCI to focus predominantly on medical management [20, 21], lacking attention to the other two domains of self-management [19, 22]. In Bangladesh, self-management promotion programs are altogether nonexistent, while previous studies have identified a need for establishing effective self-management skills to facilitate sustainable health and participation in occupation [23].

Furthermore, while much research is focused on the efficacy of self-management programs, a paucity of research remains on the perspectives of individuals with SCI and their caregivers on self-management [9, 24]. Also, no previous attempts have identified the specific self-management strategies or analyzed how these strategies work within the context of daily life among persons with SCI in LMICs, like Bangladesh. As persons with SCI self-manage not individually but together with family and/or persons in their communities [9], it is important to include others, particularly primary caregivers, to examine self-management in a manner that is inclusive of individual and comanagement skills [24]. Accordingly, there is a need for increased understanding of strategies related to the self-management of persons with SCI and their primary caregivers in LMICs, such as Bangladesh, to develop appropriate self-management programs.

### 1.1. Research Aim

This research was aimed at exploring how community-dwelling persons with SCI and their primary caregivers execute self-management strategies in daily activities in Bangladesh and at mapping these strategies to the preexisting framework in the literature.

## 2. Methods

This study was structured following the reporting guideline Standards for Reporting Qualitative Research (SRQR) (S1) [25]. The research was approved by the Tokyo Metropolitan University Research Ethics and Safety Committee (no. 19085) and the Institutional Research Review Board of the Centre for the Rehabilitation of the Paralyzed (CRP) in Dhaka, Bangladesh.

### 2.1. Study Design

A qualitative study design based on constructionist epistemology [26, 27], namely, photoelicitation focus group discussion (FGD), was used. A constructionist epistemology assumes that reality is socially constructed and that research should be conducted through interaction among researchers and participants [24]. Photoelicitation is a method of using photos that provide additive value compared to only verbally exploring participants' perceptions. Moreover, photoelicitation enhances the *phenomenological sense* through participant narrations of the photos [28]. Given the exploratory aim of this study, these photoelicitations were regarded as the most appropriate means for accessing participants' personal experiences of self-management. Through narrating experiences and sharing photographs, interaction among participants and researchers creates conditions conducive to sharing strategies and exploring each other's experiences in a more in-depth manner [29].

### 2.2. Participants and Context

Participants included individuals with SCI and their primary caregivers, selected from a database of patients discharged from CRP. CRP is the sole rehabilitation center for people with SCI in Bangladesh [30]. Two local research collaborators identified an initial pool of 50 potential participants. Inclusion criteria were as follows: adult (≥18 years) with SCI (tetraplegia and paraplegia) with at least one year of community living experience after discharge and having a primary caregiver (≥18 years) who is the main supporter/caregiver.

This study excluded persons with insufficient communication abilities and/or secondary complications that increased the risk of adverse health, such as being advised to remain on bed rest by a physician as a precaution for (worsening) pressure ulcers. Purposive sampling techniques were conducted to achieve a range of participants varying in age, gender, marital status, onset and causes of injury, and geographical locations. Research participants' demographics were matched with the national and CRP SCI demographics [31]. In Bangladesh, the sex ratio between males and females with spinal cord injury is 4.5 : 1, and the mean age of injury is 34.5 years, which is different from Western literature [32]. Along with the collaborators, the first author narrowed the pool down to 20 potential participants who were approached for the study via phone call. Fourteen agreed to voluntarily participate with their primary caregivers (see Table 1). Eight persons with SCI and one primary caregiver each formed group A. Similarly, six persons with SCI and their primary caregivers formed group B. Four participants (three “pairs” from group A and one from group B) could not participate in the second session due to personal issues (sickness, sudden family function, inability to arrange transportation, and death of a relative). The focus groups were conducted in the CRP. The recruitment procedures resulted in participants who were managing relatively well and were therefore able to be good informants, given the aim of the study.

### 2.3. Data Collection Procedures

Data collection was conducted twice in 2-hour sessions for both groups between February and March 2020. A semistructured FGD guide with open-ended questions was used for all meetings, and participants' self-selected photographs were used in the second meeting. The first and second authors, both Bangladeshi occupational therapy researchers, served as the moderator and assistant moderator. In the first session, informed consent was obtained, and participants were asked to discuss how they managed their medical health complications, emotional well-being, and roles in daily activities after discharge from hospital care (see the appendix). After two weeks, for the second session, participants brought one to three photographs that were self-captured using their smartphones and were related to daily activities, according to the instructions provided in the first session. Participants were asked why they chose those particular pictures and how the pictures connected with their management of health and daily activities. Additional probing questions included prompts related to the circumstances just described, such as triggers to their thinking, what other persons did, whether management of health and daily activities was similar or different in that situation compared to other times, and in what ways the caregivers participated. The sessions were recorded with an audio recorder (Olympus V-843), and field notes were taken to record participants' expressions, actions, and gestures.

### 2.4. Data Analysis

We used an inductive approach that primarily detailed raw data readings to derive strategies through interpretations made from the raw data. Further, a deductive analysis was utilized to compare the consistency of the data with the preceding self-management framework/model [33]. Data were analyzed following constant comparative analysis described by Bogdan and Biklen [34]. An iterative process was utilized, and emerging analytic results were undertaken to generate strategy codes, subthemes, and themes [34]. Initially, all of the focus group audio recordings (including explanations of the photographs) were transcribed verbatim. The transcripts of the first session were separately coded by the first and second authors, after which they compared their codes and discussed the similarities and the differences in order to achieve a shared coding scheme. Subsequently, the transcripts of the second session were coded by the first author and checked and refined by the second author. The comparison of similarities and differences among codes for all of the data was then conducted in a back-and-forth (iterative) process of moving between the data and codes. This coding focused on how participants managed challenges related to health and daily activities, e.g., their particular self-management strategies and the development of these particular ways of management. The coded data were then categorized through a deductive approach under medical, emotional, and role management units [14]. However, many of the codes did not fit into this taxonomy. Therefore, the remaining codes were merged into a fourth self-management component of managing social complexities. The final codes and categories were translated into English by the first author and checked by the second and third authors.

Finally, the transcripts were reviewed to verify that the code and categories reflected the actual data to ensure the quality of the analytic results through an ongoing process of meetings among the authors.

### 2.5. Trustworthiness

Several measures were taken to achieve trustworthiness following the guidelines of Santiago-Delefosse et al. [35]. Open-ended questions, field notes, and photographs were used to promote the gathering of rich data and thorough descriptions that ensured data triangulation. Independent coding of data by two of the authors further enhanced the coding quality and resulted in a robust, comprehensive analysis. Additionally during coding, the researchers used terms that closely aligned with the participants' vocabulary in order to minimize researcher bias. Prolonged engagement in the field, as well as the first and second authors being native Bangladeshis and previously affiliated with CRP, helped build trust and comfortable relationships with participants and ensured that participants' living conditions were thoroughly understood. A third-party person from CRP selected the potential participants; therefore, there was no healthcare relationship between the participants and researchers, reducing the risk of bias to the overall results. The participants in this research were no longer dependent on the CRP for their treatment, which encouraged them to talk freely and openly about their experiences. Moreover, the previous experiences of the researcher and coauthors in this research field enhanced their reflexivity. Additionally, all the authors are experienced researchers and have previously served as occupational therapists working in the rehabilitation field. The quality of analysis and emerging results were further ensured through repeated critical discussions at graduate school seminars and international conferences.

## 3. Results

Nine groups of self-management strategies resulted and were later categorized under one of the three existing components of self-management (i.e., medical, emotional, and role management) [14]; however, some of the strategy groups could not be categorized under any of these three components. Accordingly, a fourth component was identified and referred to as the *management of social complexities*. All strategies are presented in Table 2 with supporting data. The individual participants' quotes are referenced as A1–A8, and the primary caregivers as PC-A1–PC-A8 and B1–B6; PC-B1–PC-B8 represent the participants from group A and group B (introduced in Table 1). The participants' self-captured photographs were not included in this report, considering ethical issues.

### 3.1. Medical Management

#### 3.1.1. Developing and Following Consistent Routines and Habits

Participants highlighted that maintaining routine activities and habits in daily life (as advised by health professionals) and developing new routines and habits through their own designed endeavors helped prevent physical complications and facilitated participation in daily activities. Routines and habits included practicing prevention strategies, such as frequently changing posture, monitoring skin regularly, maintaining catheter cleanliness, completing physical exercises, and eating healthy, balanced diets. For example, A1 explained, in conjunction with his photograph of repositioning himself in a wheelchair:

Just after discharge from the hospital, a pressure ulcer formed on my one buttock here…changing my body positions to release pressure in 20-minute breaks became a routine, and eating nutritious food helped me to recover quickly and prevent the recurrence of the pressure ulcer, and I returned to my business.

PC-A1 also acknowledged this sentiment.

Additionally, PC-B5 mentioned:

I am used to monitoring his skin, changing his position in a timely manner, and always reminding him to avoid fried food that is not good for his health…haha [telling proudly].

Some participants mapped out their own routines and habits of managing physical complications by their own designed endeavors. This was particularly observed with participants who were living in remote areas where professional support was lacking. For example, participant B4 controlled his urinary incontinence by mapping out urination times. Other participants (A3, PC-A3, A4, PC-A4, and B1, PC-B1, and B6) worked on their medication doses according to urinary infection severity and/or improvement in symptoms after taking advice from their peers (please note that the lack of pharmacies in Bangladesh means that people usually resort to self-medication).

In other instances, participants developed personalized strategies derived from the situation. As stated by B5,

I often had tremors during catheterization. Later, I realized this was because of sleep disturbance and urinary infection… I practiced deep breathing exercises. After that, I give pressure to pee for releasing a few drops of urine for a few minutes, and then I am able to insert a catheter successfully without any complication (tremors).

#### 3.1.2. Using Health-Promoting Properties of Daily Activities

Participants stressed the use of health-promoting properties of activity engagement, such as activities that prepare the body for movement or help to prevent physical complications. For example, participants' active involvement in outdoor activities often required the movement of body parts, which indirectly acts as a strategy for preventing pressure ulcers or reducing joint pain and spasticity. B3, who runs a computer repair shop, explained a photograph of him picking an object from a high shelf in his repair shop:

I am used to always being busy repairing devices in my shop, and this task requires frequent movement of my body, which reduces the risk of pressure ulcers and other problems. When I try to pick the items from the top, I need to raise my body a little so that it releases pressure automatically.

Moreover, such engagement could also act as a form of healing. Participant A3 included a photograph of herself playing wheelchair basketball and reported that her physical condition improved while playing basketball, and she experienced minimal occurrences of complications while being involved in the sport. This engagement in daily physical activity helped her to minimize health risks.

### 3.2. Emotional Management

#### 3.2.1. Developing Authority over Feelings and Thoughts

Participants reflected that developing authority over feelings and thoughts contributed to their ability to obtain control over and cope with emotional consequences, e.g., feeling upset, anxious, depressed, and full of grief and having suicidal thoughts. One way these were overcome, with or without family support, was by engaging in recreational activities. As A1 showed his photographs of reading and writing while lying on a bed and seated in a wheelchair near the bank of the river, he explained:

I was exhausted [voice breaks at the end of sentence]!… umm… I resumed engaging in pleasurable activities, such as reading books, chatting with friends, enjoying nature, and writing my biography. These activities also inspired me because I can learn from others' struggles, and others can learn from me.

Participants also expressed more positive feelings and thoughts by visiting friends rather than staying at home for long periods of time, which increased boredom and negative thinking. Moreover, participants showed that reframing and guiding positive thinking helped them deal with their emotions successfully and facilitate daily activities. PC-A6 mentioned that this life was not an accident but a new opportunity to breathe again, and A6 noted that completing his education became a source of pride.

In addition, physical improvement provided energy for reducing stress. Participant A4, who was previously bedridden, experienced the regained ability to walk with a walker. Furthermore, performing daily activities replaced her sorrows with happiness.

Additionally, religious belief was shown to be a strong deterrent against negative emotions and a great source of mental power for participants. Participant A3 said:

If I feel destroyed, I pray to God to help me, and I believe that God has a better plan for me [gentle tone]. Believe me…Praying added extra mental strength to fight the negativity.

#### 3.2.2. Forming Inner Peace by Rewards

Rewards were mentioned either as monetary or as intangible achievements, such as earning respect, developing a sense of identity, or gaining a position in society. Participants highlighted that they had earned the rewards of being recognized for their contributions to the Bangladeshi society, which could be positive for one's emotional well-being through a sense of achievement. B4 shared through his photograph of working in a shop that he had gained inner peace from engaging in productive activities. The mental stress and negative thoughts were changed and ignored after earning profits in businesses. He stated:

I usually keep myself busy in my shop to produce more profit… in the beginning, I used to be very depressed and felt down, but now I can see how my hard work has paid off in other ways as well, neither let me feel down, by not having a single minute to think about my sorrows.

Notably, a sense of pride was established through earned rewards that also created and built autonomy, which in turn reduced emotional consequences. Participants started to respect others first, and that was then returned to them. B1 said and PC-B1 acknowledged:

I can earn 500–700 BDT per day, which some normal people cannot earn. I try to be very humble with my customers and give frequent discount offers and flexibilities. People often say that I am doing an excellent job compared to others… makes me very proud and satisfied. Now, I feel that, yes, I can do [clenches fist].

### 3.3. Role Management

#### 3.3.1. Regaining Responsibilities in Family and Society

Participants intended to restart or perform previous roles as members of their families and communities but were impaired due to the consequences of their SCIs. Also, participants created new roles and managed to return to responsibilities. A2 ran a poultry shop alone (during his father's sickness) that made a good profit and helped his mother with household chores, which he shared through his photographs that involved cleaning, folding clothes, and cutting vegetables. Similarly, B2 fulfilled the parental roles by caring for his son while his wife was at work (PC-B2). Participants managed to earn money as family heads by engaging in new, income-generating activities in light of the inability to return to the previous work. For example, B3 said, in conjunction with showing a photograph of his shop:

After the accident, I asked for money from my parents to serve my family, but… it is better to die than to depend on others [paused while looking at his feet]… I began vocational training and later started a shop with help from my family. Now, I can earn enough to serve as a family head and also support my parents.

Moreover, other social responsibilities that participants noted are related to connecting with relatives and other members of the community, which they regained through use of social media and mobile phones. Participant A4 shared that she acquired updates on the health of her sick grandparents via phone calls instead of in-person visits.

#### 3.3.2. Engaging in Charitable Activity

Engaging in charitable activities was useful to participants for fulfilling their duties to mankind as social beings. These activities introduced additional roles that were not mandatory but helped participants to become role models in a society where they are considered incapable. Charitable activity was established by helping others by offering financial or educational support and by providing facilities for treatment information. In return, participants gained the respect of others. A2 shared:

Once, when I applied for a disability card, I saw a young boy with a SCI who was helping other disabled persons, including me…then, I decided to do something for others. Later on, I created a Facebook group where we can share support, advice, and information with other persons with disabilities.

Likewise, A5 helped peers in remote areas by sending wheelchair parts for repair. Also, participants assisted with social services by raising awareness of the prevention of SCI, especially from scarf-related injury (injury to the neck when women's head scarves get stuck in rickshaw motors). Such significant activity was very motivational for continuing on with SCI. B2 said:

I have a special discount (5%–10%) for disabled persons in my shop [smiling with shiny eyes]. I feel proud of this. I think it will help to support them as well as inspire more normal persons to do that.

### 3.4. Management of Social Complexities

Management of social complexities refers to the management of deteriorating relationships among closely bonded people and the social consequences faced after an injury. Participants noted that their actual lives started again after they returned to society, but they often had to deal with conflicts, such as being dispossessed from an inherited property or having a partner who wished to divorce them because they had become a burden. Moreover, social stigmatization, inaccessibility of the environment, and abandonment were very prominent in their experiences. Participants managed these difficulties by either relocating themselves to friendlier environments or by making other people become friendlier in their environment by using dynamics such as modesty, incorporation of skills, and advocacy.

#### 3.4.1. Relocating to Another Environment

Relocation was evident, as some participants moved to a more disability-friendly environment to escape destitution, negative attitudes, and discrimination from society, which had previously prevented their participation in daily activities. A5 stated:

When I was running a shop in my village, most of the people called my shop “Lengrardokan” (Lame's shop), and they would sometimes throw small stones on my shop roof. It was unbearable, it hurt me, and it was impossible to stop their mouths and behavior [sad face and swallowing frequently]. I moved to another place far away from them after closing the shop but maintained a good relationship with all.

Most of the participants who could not bear to be around those negative attitudes relocated to a comfortable place in an urbanized area where education and awareness of people with disabilities were not as significant of issues, and transportation systems were better compared to rural areas. A4 said:

People always stared at me as if I were an animal in the zoo [sigh]. Also, I gradually felt like I was becoming a burden to my loved ones, so I moved to the city area near the hospital (CRP) so that I could stay in an area where other persons with SCI live and could also continue my treatments.

#### 3.4.2. Behaving in an Assertive Manner

Participants expressed assertiveness to society by creating positive perceptions toward individuals with SCI or other disabilities. Some participants discussed experiences where they felt compelled to argue with their close ones for their interests, which was beyond maintaining family responsibilities. For example, B1 said:

After the accident, our property had to be divided among me and my brothers and sisters. My mother asked my brothers to give some extra land to me, but they became angry and refused to divide it equally because they had already donated a huge amount to my treatment costs [removed eye contact and looking toward the floor]. I asserted my need for land to make money for my survival [continued eye contact]. They acknowledged this and agreed to divide the properties equally.

As another example of dealing with ongoing social issues, some participants preferred to change the perceptions of others by serving as examples of the capabilities of individuals with SCI or by explaining the kinds of issues they deal with in daily life (A2, PC-A3). Participants managed social negligence by proving their worth. B4 shared a photograph of a visit to the university campus, and PC-B4 agreed:

I used to visit the university campus to eat some traditional foods, but the restaurant employee stopped me from entering because he assumed that a person in a wheelchair would be a beggar. After I paid the bill, including his extra tip, he seemed to feel guilty. Now, if I visit there, they warmly welcome me [smile].

#### 3.4.3. Advocacy for Social Changes

Participants evoked their advocacy for social changes by promoting or defending access to facilities as well as their rights to be included in the mainstream of society so that they could engage in daily activities. Participants often faced barriers to participating in daily activities due to the inaccessibility of the environment and to the violation of social rights. For example, as participant PC-B3 shared together with B3:

Once, we purchased a museum ticket, and the staff overlooked the (disability) discount ticket price. We objected to such attitudes from governmental staff and complained to the authorities. Finally, they started to consider a 50% discount price for persons with disabilities [loud voice with confidence].

Participants could also use the support of organizations because it is difficult for individuals alone to advocate for necessary changes. For example, A5 and PC-A5 explained, with a photograph of a ramp:

Once, we went to a shopping mall nearby that had newly opened, but there was no ramp to enter…then, after informing CRP, we went there to talk to the owner along with the staff of CRP…they later made a ramp to the shopping mall [victory smile].

## 4. Discussion

This research explored how persons with SCI and their primary caregivers execute self-management strategies in daily community life and then mapped these strategies to a leading, preexisting self-management framework. Categorizing the nine identified self-management strategies challenged the current composition of the framework of self-management, which is composed of the medical, emotional, and role management categories. Instead, some strategies observed in this study indicated that managing social consequences is equally essential. These strategies transcended the role management component, given the sociocultural context of a LMIC, like Bangladesh. These appeared to be even more important among participants living in remote areas. Moreover, we note that these strategies are all but reported in the self-management literature that is predominantly from high-income countries. These findings will be discussed next, highlighting the complexity in nature and interconnectedness of the components of self-management in daily life.

Findings of the medical management strategies appeared similar to those reported in the international literature for persons with SCI, in that medical problems are reduced by controlling or preventing diseases, creating new health behaviors, and personalizing prevention strategies [36–38]. These findings call into question the manner in which the research participants achieved such a feat in a lower-middle-income country such as Bangladesh. Based on participant characteristics, we conclude that many participants could be considered long-term survivors within their social context, whereas most Bangladeshi persons would perish within ten years of acquiring a SCI. What stands out in the data is their experiences of resourcefulness and their engagement in valued daily activities as they pivot toward deploying strategies intentionally and unintentionally, such as discovering adequate medication dosages or engaging in valued activities. What remains a question for further study is why the participants in this study appeared successful at managing their health compared to the majority of persons with SCI in Bangladesh. Clarifying the interplay between health status and participation in daily activities should yield necessary, valuable knowledge with regard to formulating effective strategies for self-management of health.

Participants exhibited various coping strategies for emotional distress, most of which were analogous to the previous literature in the field (e.g., developing authority on emotional issues) [12, 39]. Importantly, engaging in income-generating activities resulted in the management of emotions. The severe sociocultural conditions of the participants may have made the importance of this strategy more pronounced. Furthermore, religiosity was also understood as a powerful strategy, and Islamic beliefs served as sources of consolation in terms of acceptance of reality and facilitated actions to improve daily life, as is also reported in the literature [39, 40]. On the other hand, religious beliefs seem to be utilized less frequently in Western nations [41]. Therefore, the importance and efficacy of both income-generating activities and religiosity should be studied in greater depth to gather more information on the best possible strategies for managing emotions.

The management of roles was understood as the regaining of previous roles and development new roles; for example, engaging in income-generating activities resulted in the regaining of the head of the family role, which was the main focus of the male participants. Traditionally, Bangladeshi males are primarily responsible for financially supporting their families, while females are mostly the homemakers. However, the example of participant B3 showed that these role patterns are not set in stone. In addition, other participants shared role responsibilities with family members and society [42]. A new insight to the international literature on self-management strategies was the development of a noble role by engaging in charitable activity. Traditionally, persons with disabilities have been more likely to be viewed as subjects receiving charity from others. However, these research participants afforded their charity to others. Whether this is a cultural or religious dimension specific to Bangladeshi context or unique to these research participants with SCI should be further studied. Either way, the finding adds a new, possibly efficacious, strategy for self-management to the existing array of strategies.

In particular, a new contribution of this study is the component of *management of social complexities*. This finding challenges current descriptions of self-management [14, 43], none of which addresses this component. Further research should consider whether this is an anomaly of most studies focusing on medical consequences [20]. Also, this new component could be the result of the myriad of socioeconomic and cultural conditions of the participants, which also appeared to pose more severe challenges compared to challenges of those living in higher-income countries, given the poor educational and awareness levels of the general population toward people living with disability [2, 42, 44]. However, these challenges posed fewer problems in urban areas, particularly in neighborhoods near the CRP, where the institution and people in wheelchairs are commonplace. This helped to nurture more inclusive communities with more accessible structures that not only promoted daily activities but also raised the chances of individuals with SCI becoming a part of mainstream society [45]. Another strategy for increasing inclusion was managing society through assertive manners and utilizing self-advocacy for social rights. These strategies are aimed at changing people's perceptions wherein participants created space for their own inclusion in society. Culturally, assertive manners displayed with a modest attitude are more likely to be positively received and acted upon by others. However, assertive manners can also be necessary to have one's rights respected [9].

The results of this study also show the complexity and interconnectedness of each component of self-management that the literature explains separately. We found that the “earning by doing” strategy prevented complications and optimized physical health, managed emotional consequences by connecting the past and present (emotional), reemerged the family head roles that facilitate to run the joint family (role), and yielded social value by showing dynamic responses in reciprocity when in connection with others (social complexities). Arguably, earnings, especially in the Bangladeshi context, are the reason these participants survive, as the income perhaps affords them to buy resources such as cushions and catheters. This, in turn, helps to facilitate the comprehensive self-management of health and daily activities. Finally, this critical result supports the notion that focus on individual health, emotional well-being, and roles is insufficient, and self-management hinges on tackling social problems that constrain the health and participation in daily activities of persons with SCI equally, at least in a lower-middle-income country, like Bangladesh [23].

### 4.1. Strength and Limitations of the Study

The strength of this study was the heterogeneity of participants, reflecting the population of people with SCI in Bangladesh. They were resourceful and shared diverse experiences/strategies that enabled them to survive longer and manage their daily activities well in comparison to most people with SCI in Bangladesh. Another strength was the inclusion of primary caregivers, which facilitated data gathering on comanagement skills that were deemed essential in the local context. Also, combining discussion with photoelicitation added credibility to the study, as the participants' recollections were stimulated by visual clues in addition to their conversations. As a result of these strengths, existing notions of self-management could be challenged, and an additional, interconnected self-management component was identified. On the other hand, the inability to discuss the management of sexual problems, as the focus groups consisted of a mix of married, single, and male and female participants, some of whom participated with a parent as a primary caregiver, was a limitation. Photoelicitation may have further led the participants to refrain from sharing such private issues, which, as in many cultures besides Bangladeshi culture, is a topic people do not discuss publicly. Future individualized interviews may explore this issue. The sampling technique was conducive to recruiting participants who could share good insights relevant to the research topic; however, this study sample likely overrepresented long-term survivors who may be better at self-management in daily life than most persons with SCI, who arguably self-manage poorly [5].

### 4.2. Implications and Recommendations

This study adds knowledge to the literature that should be heeded by healthcare professionals when considering the need for community (peer) support programs. The strategies identified in this study and other studies [23] can be used when persons with SCI struggle with adverse conditions. Peer and local community support is particularly critical when regular follow-up visits by professionals are limited due to institutions lacking the human and financial resources as well as the lack of local affordable health and welfare services [46]. Moreover, the newly identified component, *management of social complexities*, will help healthcare professionals, especially occupational therapists, focus not only on the individual's health problems but also on additional social problems to facilitate social justice in daily life [10]. Recent local development of a community outreach program for persons with SCI failed to reduce comorbidity and mortality [47]. In the light of this study, we wonder whether the study failed because its main medical focus was limited to prevention of secondary complications and it did not address the social, economic, and cultural determinants of health and participation in daily activities. Therefore, it is recommended that any self-management enhancement program should integrate all four components. Accordingly, the results of this study may also inform the future development of self-management enhancement programs to better support the daily life activities and health of persons with SCI living in the community.

## 5. Conclusion

This study contributed to knowledge and understanding of the complexity of self-management and socially contextualized strategies. In turn, this may guide the development of a community support program for persons with SCI in Bangladesh and beyond. Including a newly uncovered fourth component of *management of social complexities* of self-management reinforces the importance of understanding and acting upon individual and societal perspectives in the local context, which may or may not be more pressing in a lower-middle-income country, like Bangladesh, compared to higher-income countries.

## Figures and Tables

**Table 1:** Participant demographic characteristics.

Participants	Gender	Age	Education level	Marital status	Time since injury (years)	Causes of injury	SCI level	Severity of injury	Occupation (IgA)	Mobility device	Living area	Participants (primary caregiver, age)
A1	M	31	S	Married	7	RTA	C5–6	Incomplete	No	W/C, crutch	Rural	Mother (50)
A2	M	30	P	Single	16	RTA	T7–12	Complete	Poultry shop	W/C	Semirural	Mother (50)
A3	F	18	P	Single	3	FFT	Coccyx (S4–5)	Incomplete	No	W/C, W/F	Urban	Mother (35)
A4	F	21	S	Single	2	Fall of heavy object on back	T10	Complete	No (basketball player)	W/C	Urban	Brother (32)
A5	M	32	S	Single	26	Tuberculosis	T11	Incomplete	Business with friend	W/C	Rural	Sister (22)
A6^∗^	M	40	S	Married	20	Gunshot	T12	Complete	Shop	W/C	Urban	Wife (27)
A7^∗^	M	50	P	Married	12	FFT	T	Complete	No	W/C	Rural	Wife (45)
A8^∗^	M	24	P	Single	17	Fall of heavy object on neck	C4	Complete	No	W/C	Rural	Mother (45)
B1	M	38	P	Married	13	FFH	T12	Complete	Elec. shop	W/C	Rural	Wife (27)
B2	M	33	S	Married	6	RTA	T12	Incomplete	Online business with shop	W/C	Semirural	Wife (24)
B3	M	30	D	Married	4	FFT	T7	Complete	Computer/mobile servicing	W/C	Urban	Wife (25)
B4	M	27	S	Single	15	FWCLH	T9	Complete	Grocery shop	W/C	Semirural	Mother (60)
B5	M	34	G	Single	9	FFH	C4	Complete	No	W/C	Semirural	Paid carer (27)
B6^∗^	M	31	P	Married	3	RTA	T7	Complete	No	W/C	Semirural	Wife (27)

A1–8 participants are from the first group (A), and B1–6 participants are from the second group (B). ^∗^Participants that could not attend the second session of the focus group discussion. M: male; F: female; S: completed secondary school; P: completed primary school; D: diploma (short course on a subject); G: graduate bachelor course; RTA: road traffic accident; FFT: fall from a tree; FFH: fall from height; FWCLH: fall while carrying heavy load on the head; IgA: income-generating activities; W/C: wheelchair; W/F: walking frame.

**Table 2:** Themes, categories, codes, and supporting data.

Themes	Categories (subthemes)	Codes	Meaning bearing units
Medical management			
Developing and following consistent routines and habits	Maintaining prevention strategies	Using existing healthcare strategies following advice	Cleaning and changing the catheter regularly following advice from doctors (A2, B1, A7, and B6) Using a newspaper on top of a cushion and washing hands and clothes to keep things clean (A2, B1, A7, and B6) Changing sitting posture, therapies for back pain (A1) Doing pressure release every 20 min (A1, A5, B2, B5, A3, B4, and PC-A1) Not putting pressure on one (skin) area (B2) Changing body position frequently by transferring to other seats/beds (A1, PC-A1, and PC-B5) Monitoring skin regularly to prevent pressure ulcers (A5, PC-B5) Stretching and doing other exercises (A1, PC-A1, B3, PC-B3)
	Following a balanced diet	Eating nutritious food Avoiding unhealthy food	Bowel-bladder incontinence and infections were prevented by the following: Eating healthy and nutritious food (A1, A5, B5, and B6) Avoiding oily food and junk food (A1, A5, and PC-B5) Having home-cooked foods (PC-A1) Drinking plenty of water regularly (A2, A5, and B1) Taking food in a timely manner and in small portions (A2)
	Adopting strategies from trusted laypersons	Using medication following peer strategies	Taking antibiotics to obtain urine control, as advised by familiar persons (A4) Learning first aid management strategies from peers to be healthy (A3)
	Learning from bad experiences	Preventing recurrence	Using an additional cushion on a wheelchair seat after experiencing pressure ulcer while playing basketball (A5)
	Self-trial toward mastery	Self-prescription Trial	Taking medicine for urine infection based on the consultation with a lay relative in lieu of a physician (A3, PC-A3) Changing medication dosage for urine infection following own judgments according to improvements (A4, PC-A4, PC-B1, B1, and B6)
	Discovering new habits	Self-derived ideas New ways	Creating a routine urination time to prevent incontinence due to inability to hold urine (B4) Discovering the use of gel to prevent bleeding during catheterization (A4) Finding out the way (breathing, waiting, the pressure to pee, and catheter usage) to manage tremor during catheterization (B5)
	Solving problems	Dressing at home Taking vitamin C	Dressing the pressure ulcer regularly at home because the hospital is far (B2, PC-B2). Drinking lemon juice for pressure ulcer recovery (B2)
Using health-promoting properties of daily activities	Active involvement in outdoor activities	Engaging in paid and unpaid work Playing basketball Doing a physical activity that influences physical improvement	Working in a computer/mobile phone repair shop requires frequent movement of the body (B3) Doing business actively reduced the risk of pressure ulcers (B2, B3) Removing objects from the top place helped release pressure from the buttocks (B3) The routine of transferring to the vehicle to buy goods for stocking the shop improved physical condition (A5) Going outside and riding in a vehicle minimized physical problems (A5) Playing basketball decreased physical problems and improved balance (A3) Doing exercise for physical improvement (required for returning to a job) reduced the risk of secondary complications (B3)
Emotional management			
Developing authority over feelings and thoughts	Engaging in recreational activities	Participating in meaningful tasks Recreation	Resuming life while upset and depressed through browsing the Internet (A1, A3, B4, B3, and B5), using social media (A1, A2, A3, A4, A5, B1, B2, B3, B4, and B5), watching television (B2, B5), chatting with friends (A1, A3, and B5), reading books and writing an autobiography (A1, B5), listening to music (A3, B2), playing mobile games (A1), and traveling for sightseeing (A1, B5)
	Creating a positive environment	Changing situations Avoiding unwanted moments	Going outside from home, where boredom and negative thinking were increased (A2) Spending time alone away from all (A2) Leaving the situation that created anger (B1)
	Positive reframing	Positive thinking Having faith in self Setting an action plan	Thinking positively and making a fresh start against feeling depressed (A3, PC-A3) Having faith in oneself for the future (A3) Realizing that this is not the end of life (A4) Eagerness to get back to studying (A4, A6) Learning to be skillful in a wheelchair (A2) Accepting the truth and planning for the future (PC-A6)
	Self-guidance	Acceptance Comparing with peers Not feeling different	Admitting the consequences as natural and going with the flow (A1, PC-A5) Explaining to self not to let the sorrows take control over their capacities (A5) Convincing one's mind that accidents happen (B2) Gaining mental strength through seeing others in the same condition (A1) Not feeling alone and taking inspiration from others with disabilities (B1)
	Spiritual healing	Religious belief	Praying to God adds extra mental power to fight negativity (A3, B1, and A4) Believing that God has a better plan (A1, A3, and PC-B4)
	Family love and support	Encouragement of life Motivating to move on Being supported by close ones	Giving up committing suicide and thinking of wife's and son's futures (B3) Arranging a tour by the family to cheer up the mind (B5) Staying at one's side and being consoled in tough times (A1, PC-A1) Being encouraged to move ahead (A2)
	Physical improvement facilitated happiness	Hope for becoming independent Inspiring themselves to be active	Walking with frame replaced sorrows with happiness and inspiration for trying more (A4) Improving balance brings a genuine smile to the face (B3)
Forming inner peace by rewards	Business in productive activity	Business Spending quality time	Being busy in the shop gave the feeling of being a productive person and to never think about sorrows (B4) Passing enjoyable moments in the shop because many people gather and chat (B1) Spending time in the shop and producing income fight depression (B2)
	Sense of pride	Being a topper Employment	Becoming a top basketball player with remunerations improved confidence and willpower (A3) Earning money equal or higher than that of normal people makes one proud and satisfied (B1, PC-B1)
	Autonomy	Independence Social identity Achievement	Serving the family improved independence (B4) Family and society gave importance to one's opinion (B4) Happy to gain a respectable social identity through running the shop (B2) Achieving many things with a disability-inspired life (A2)
Role management			
Regaining responsibilities in family and society	Contributing roles dynamically	Fulfilling family role Becoming an advisor Shifting roles	Managing father's business during sickness (A2) Helping mother with household chores (A2, A4, and A3) Managing household work alone while the mother is at her job (A4) Taking care of the child and home while the wife is at her job (B2, PC-B2) Reducing the burden on a family member by helping oneself (A1)
	Earning for the family through income-generating activities	Being head of the family Serving the family Relying on self Building a career	Starting a poultry shop with good profit (A2) Starting a shop after vocational training to serve as the head of the family (B1, B3, and B4) Getting remuneration by playing basketball (A3) Building up a career (A3, B1, B2, B4)
	Being connected with others in any situation	Maintaining social connection	Talking over the phone instead of visiting sick elderly grandparents (A4) Chatting with friends on social media to not lose them (B5) Going sightseeing with old friends (B4) Connecting with relatives via the Internet (B1)
Engaging in charitable activity	Helping others in need	Providing unconditional help	Repairing other persons' wheelchairs without charge (A5) Sending required items to peers in remote areas (A5, B1) Advising peers living in remote areas (A5, B1) Providing information and education to peers through a Facebook group (A1, A2, and B2)
	Delivering social service	Giving a special discount Raising awareness about accidents	Providing special discounts to disabled clients in the shop (B2) Raising awareness among people about spinal cord injury, especially scarf injury (B1)
Management of social complexities			
Relocating to another environment	Moving to a comfortable place	Moving to another place Maintaining distance	Moving to a city area and creating a new start to avoid negative social attitudes (A3, A4, and B3) Shifting to another place and keeping a good relationship with close people from a distance (A5)
Behaving in an assertive manner	Handling conflict with close people warmly	Emotional attachment	Convincing close people by asserting their need and using emotion when being deprived from usual support (B1, B2, and B3) Showing modest behavior to gain empathy (B1, A1, and A5)
	Dealing with social stigma	Changing perceptions	Giving a successful example of another person with SCI (A2, PC-A3) Explaining the pathogenesis and prognosis of SCI to the society (A2, B2, B4, and A4)
	Resigning to deprivation	Resource utilization Compensation	Asking for help from traffic police to get on public transport when the driver denied (A5) Continuing to study at home due to inaccessible toilet at the school (A5, B4)
	Proving self-worth against social negligence	Building capability	Changing the attitude of a restaurant owner through paying the bills and giving tips (B4, PC-B4) Showing the capability of living without support (B2)
Advocacy for social change	Stressing accessible environment	Ensuring accessibility	Asking the owner repeatedly for a ramp to the house (PC-A5) Campaigning for an accessible entrance to the shopping mall (B1, A5, and PC-A5) Raising awareness to build a ramp and elevator in a newly constructed building (A2, B2)
	Claiming human rights	Disability rights	Asking for and raising awareness about disability special discount tickets and services in the museum (B1, B5, PC-B3, and B3) Ensuring disability priority service in the hospital and public places (B2)

PC: primary caregiver.

## Data Availability

The participants' data used for the findings of this study are included within the article, except for the participants' photographs, in consideration of ethical issues. For more information, contact the corresponding author.

## Appendix

### Questionnaire to Guide Focus Group Discussion

The initial question:

To begin with, it is good to know some things about your daily lives. Can you please tell us about them?

- For the person with SCI:
- What does your daily life look like? For example, describe what you do on a typical day. How do you pass your productive time, and how do you pass your leisure or resting time? Why so? Why do you use different ways/methods to manage your health/daily life?
- What challenges have you faced since discharge from the rehabilitation center and now? Can you please share your short story or experience with the challenge?
- How did you manage your health challenges? Why so? How about other challenges? How have other participants managed those challenges?
- We know that chronic health-related complications and long-term disabilities interfere with your emotional well-being. Therefore, how did you manage or control your emotions during illness or any social conflicts (if you ever experience them)?
- What other challenges have you faced living in the community? Can you please share the story? If so, how did you manage these issues in your living area? If you could not manage, then what did you do in such a situation?
- How did you come up with ideas to manage the challenges you had, or how did you bring the thinking of managing your challenges in that way? What do you think? How is your daily life a bit different from that of other participants?
- What do you think? How could you manage better than the way you are managing now? How could you overcome challenges that you are now facing but cannot manage very well? What would you recommend to each other to manage these challenges in a better way? What do you think? Which things can help you to manage your challenges better?
- For primary caregivers:
- What does your daily life look like? For example, describe what you do on a typical day. How do you feel about your daily life including a person with SCI? Why so?
- How have you managed his/her health complications, recreate new or old roles, and navigate emotional challenges since discharge from the rehabilitation center? Can you please tell us the story? What were your feelings at that moment? Why so? Do you have any other experiences to share?
- How did you contribute to managing the daily life of your persons with SCI? Why so? What about the experiences of other caregivers? Would you like to add another significant contribution of yours?
- What other major problems have you faced after having a SCI? Why did those problems occur in your situation? How did you manage those problems or challenges? Why so?
- How did you come up with ideas for managing the challenges you had, or how did you bring the thinking of managing your challenges in those ways? What do you think? How is your daily life a bit different from that of other participants?
- What do you think? How can challenges be managed better compared to how you are managing them now? How could you overcome the challenges that you are facing now but cannot manage well? What would you recommend to other persons with SCI for managing these challenges in a better way? What do you think could help you to better manage your challenges?

## Supplementary Materials

Supplementary Materials See the table in this section for the detailed checklist for the reporting guideline Standards for Reporting Qualitative Research (SRQR).
